# COVID-19 mortality attenuated during widespread Omicron transmission, Denmark, 2020 to 2022

**DOI:** 10.2807/1560-7917.ES.2023.28.3.2200547

**Published:** 2023-01-19

**Authors:** Nikolaj U Friis, Tomas Martin-Bertelsen, Rasmus K Pedersen, Jens Nielsen, Tyra G Krause, Viggo Andreasen, Lasse S Vestergaard

**Affiliations:** 1 Epidemiological Infectious Disease Preparedness, Statens Serum Institut, Copenhagen, Denmark; 2 PandemiX Center, Department of Science and Environment, Roskilde University, Roskilde, Denmark

**Keywords:** SARS-CoV-2, COVID-19, Omicron, mortality, surveillance

## Abstract

**Background:**

It sparked considerable attention from international media when Denmark lifted restrictions against COVID-19 in February 2022 amidst widespread transmission of the new SARS-CoV-2 Omicron variant and a steep rise in reported COVID-19 mortality based on the 30-day COVID-19 death count.

**Aim:**

Our aim was to investigate how coincidental infections affected COVID-19 mortality estimates following the introduction of the Omicron variant in late 2021.

**Methods:**

We compared the 30-day COVID-19 death count with the observed mortality using three alternative mortality estimation methods; (i) a mathematical model to correct the 30-day COVID-19 death count for coincidental deaths, (ii) the Causes of Death Registry (CDR) and (iii) all-cause excess mortality.

**Results:**

There was a substantial peak in the 30-day COVID-19 death count following the emergence of the Omicron variant in late 2021. However, there was also a substantial change in the proportion of coincidental deaths, increasing from 10–20% to around 40% of the recorded COVID-19 deaths. The high number of 30-day COVID-19 deaths was not reflected in the number of COVID-19 deaths in the CDR and the all-cause excess mortality surveillance.

**Conclusion:**

Our analysis showed a distinct change in the mortality pattern following the introduction of Omicron in late 2021 with a markedly higher proportion of people estimated to have died *with,* rather than *of,* COVID-19 compared with mortality patterns observed earlier in the COVID-19 pandemic. Our findings highlight the importance of incorporating alternative mortality surveillance methods to more correctly estimate the burden of COVID-19 as the pandemic continues to evolve.

**Key public health message**

**What did you want to address in this study?**

When Denmark in February 2022 lifted all restrictions against COVID-19 amidst wide spread of the new Omicron variant, it looked as if the number of COVID-19 deaths rose steeply, when in fact, most of the deaths were due to something else but those dying happened to also be positive for COVID-19 at the time. We set out to find out how many recorded deaths were actually due to COVID-19.

**What have we learnt from this study?**

We compared our normally used 30-day COVID-19 death count against alternative mortality surveillance methods, including the national Causes of Death registry, all-cause excess mortality, and a mathematical model to distinguish those who died *with* COVID-19 from those who died *of* COVID-19. This showed that during periods of widespread virus transmission, it is essential to separate dying *with* and dying *of* COVID-19 to tell the mortality burden correctly. We also show how COVID-19 mortality was lower when the Omicron variant circulated.

**What are the implications of your findings for public health?**

Different mortality surveillance methods can supplement each other to estimate more correctly the mortality impact and hence the public health burden of COVID-19, and disease in general, ensuring more correct and unbiased information to guide public health authorities, media and the general public.

## Introduction

By early February 2022, almost all societal measures and restrictions implemented against coronavirus disease (COVID-19) in Denmark were lifted. It happened at a time of record high case numbers following a recent and rapid spread of the emerging severe acute respiratory syndrome coronavirus 2 (SARS-CoV-2) Omicron variant (Phylogenetic Assignment of Named Global Outbreak (Pango) lineage designation: B.1.1.529), which proved more transmissible [1] but less virulent [2,3] than previous variants of the SARS-CoV-2 virus. This policy change sparked considerable international attention [4,5], in particular with regards to the seemingly steep rise in reported COVID-19 mortality which, at the time, was based on the 30-day COVID-19 death count [6].

Determining COVID-19 mortality is not straightforward. The varied and often unspecific symptoms may make it difficult to establish if SARS-CoV-2 infection was indeed the main cause of death, particularly in the elderly population with often multiple underlying diseases. Even if a deceased person has tested positive for SARS-CoV-2 before death, the infection may in some cases be coincidental. Therefore, the question of how to establish and quantify the real burden of COVID-19 mortality has been widely debated throughout the pandemic. An alternative method to estimate the burden is to quantify COVID-19-related mortality mathematically rather than by identifying exactly the individuals that died from the infection. Such an approach will be similar to the modern analysis of the burden of influenza [7,8]. The Danish surveillance system provides three data sources to describe the mortality burden of COVID-19: (i) automated register-based surveillance, (ii) individual death certificates and (iii) deviations in all-cause mortality patterns.

Acknowledging that the 30-day COVID-19 death count could be misleading, our aim was to investigate how coincidental infections affected COVID-19 mortality estimates following the introduction of the Omicron variant in late 2021. Here, we provide an in-depth analysis of the mortality burden of Omicron by comparing the 30-day COVID-19 death count to the observed mortality pattern using three alternative surveillance methods; (i) a model created to correct the 30-day COVID-19 death count for coincidental deaths, (ii) the Causes of Death Registry (CDR), and (iii) all-cause excess mortality.

## Methods

### 30-day COVID-19 death count

Right from the onset of the pandemic, the national COVID-19 surveillance system has been recording all deaths in the Civil Registration System where date of death occurred within 30 days of the primary positive SARS-CoV-2 PCR test result of an infection episode. This 30-day COVID-19 death count is generated automatically from electronic registers for the purpose of real-time surveillance.

### Model to correct the 30-day COVID-19 death count for coincidental deaths

At the peak of COVID-19 transmission, in mid-February 2022, the proportion of the population in Denmark who had tested positive within the preceding 30 days had increased to 22%. Hence, the risk of coincidental deaths also increased substantially. From all-cause mortality and reported cases, we can calculate the expected proportion of incidental deaths and, in turn, the proportion of deaths that are attributable to COVID-19. Similar calculations have been discussed elsewhere [9].

The calculation is carried out as follows:

Similar to the approach for estimating influenza excess mortality, we consider all-cause mortality, D, as the sum of those deaths that would have occurred in the absence of COVID-19, X, and those deaths that were caused by COVID-19, Y. Here, Y includes deaths where COVID-19 is the direct cause of death as well as deaths where COVID-19 is only a contributing factor. Thus, X and Y are statistical (or population level) concepts and in particular, Y accounts for deaths directly attributable to COVID-19 as well as for the increased mortality among persons with underlying severe illness.

The probability of having tested positive within the previous 30 days, we denote P. Note that P is the proportion of the population that has tested positive within the previous 30 days and not the probability of a test being positive. Thus, P comes directly from surveillance data and is not an unknown quantity requiring estimation. Deaths unrelated to COVID-19, X, will also occur among those who have tested positive for COVID-19 within the last 30 days. For such unrelated deaths the probability of dying is independent of the probability of testing positive, which implies that the product PX is the number of COVID-19-registered deaths among unrelated deaths. As discussed in the following section, very few COVID-19-related deaths are not detected in the Danish surveillance system. For this reason, we assume in our calculations that all COVID-19-related mortality has been identified. Thus, the total COVID-19-registered deaths, C, is the sum of Y and PX.

**Figure 1:**
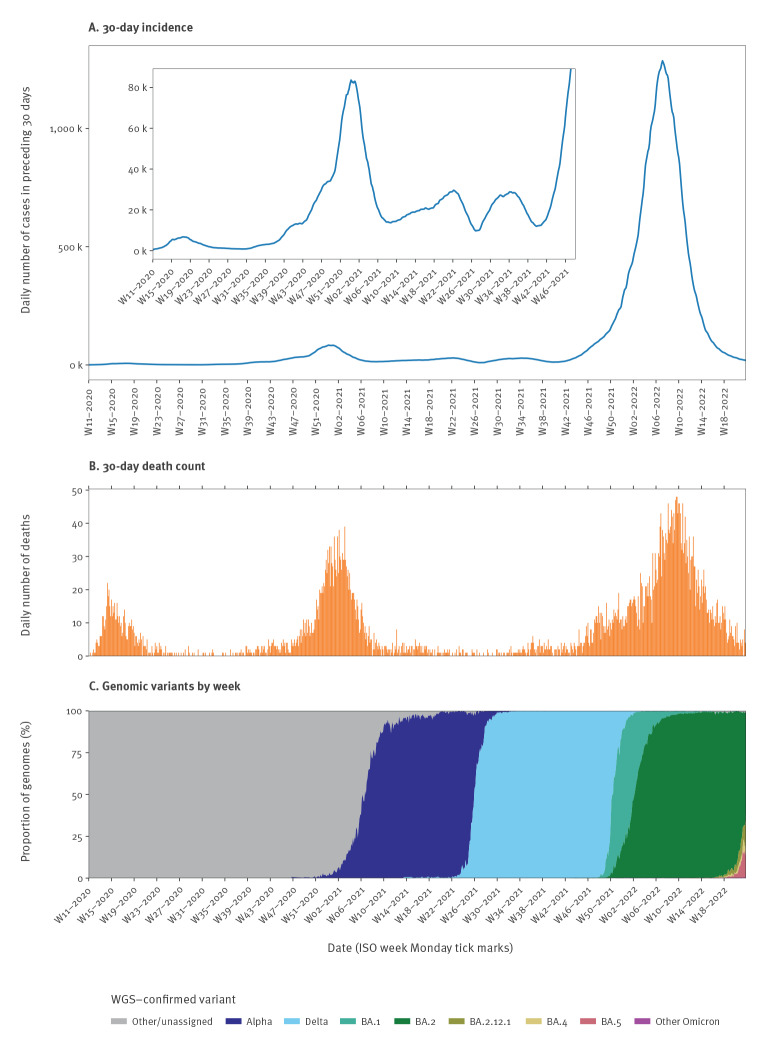
30-day COVID-19 case incidence and death count^a^ by day, and weekly SARS-CoV-2 genomic variant^b^ distribution, Denmark, week 11/2020–week 21/2022

COVID-19: coronavirus disease; SARS-CoV-2: severe acute respiratory syndrome coronavirus 2; WGS: whole genome sequencing.

^a^ Including deaths with and of COVID-19.

^b^ All variants denoted ‘BA.(x)’ are sublineages of Omicron.

At the start of 2022, testing activity and incidence in Denmark was high as seen in the large number of positive cases shown in Figure 1A. Thus, P was large in that period, as discussed in the previous section, and consequently PX must also have been high at this time. Since D, C and P are known, it is possible to estimate X and Y as X = (D − C)/(1 − P) and Y = (C − PD)/(1 − P). This follows directly mathematically from the definitions of C and D.

Since both COVID-19 prevalence and mortality are age-dependent, the estimation was carried out in age groups 0–19, 20–39, 40–59, 60–69, 70–79 and ≥ 80 years. For ages 0 to 59 years, the estimate of unrelated mortality with COVID-19-diagnosis, PX, occasionally exceeded registered COVID-19 mortality, C. While this could be due to unregistered COVID-19 deaths, the low mortality in these age groups suggests that statistical fluctuations have an effect (see Supplement). In these cases, all registered mortality in the age groups counted as unrelated. This has little impact on the total estimate, as the bulk of mortality (COVID-19 and other deaths) usually occur in older age groups. For brevity, we omit the details of the statistical considerations of the model to correct for the 30-day COVID-19 death count for coincidental deaths here but include the details in the Supplement.

### COVID-19 mortality based on death certificates

We analysed death certificates from the national CDR, which includes all deaths in Denmark. After medical assessment, the Danish Health Data Authority validates cause of death certificates. Every death for which one of the ICD-10 codes listed in the Table were registered as an 'underlying cause of death’ were included as a death of COVID-19 based on validated death certificates. Certificates that exclusively listed COVID-19 as a ‘significant condition contributing to death’ were not included. This is in accordance with guidelines from the World Health Organization (WHO) [10]. The codes U07.1 and U07.2 are the WHO-recommended emergency use ICD-10 diagnostic codes for COVID-19 [10]. B.34.2A and B.97.2A are additional codes regularly in use in Denmark that have been found to have a positive predictive value for COVID-19 of 99% [11].

**Table:** ICD-10 codes used to capture COVID-19 deaths in the Causes of Death Registry, Denmark, 2020–2022

ICD-10 code	Description
B.34.2	Coronavirus infection, unspecified
B.34.2A	COVID-19 infection, location not specified
B.97.2A	COVID-19, acute respiratory syndrome
U07.1	COVID-19, virus identified
U07.2	COVID-19, virus not identified

COVID-19: coronavirus disease; ICD: international classification of diseases.

### Excess all-cause mortality

Estimation of excess all-cause mortality in the population is another approach to gauge the mortality burden of a public health event such as the COVID-19 pandemic. This estimation relies on the observed weekly numbers of deaths from all causes and calculating the excess (deviation from historical baseline) using the EuroMOMO statistical algorithm, previously described elsewhere [12].

## Results

### 30-day COVID-19 death count

The total number of 30-day COVID-19 case fatalities in Denmark from the onset of the pandemic until 29 May 2022 was 6,363. However, the daily 30-day COVID-19 death count fluctuated considerably during the different waves of SARS-CoV-2 transmission (Figure 1B). Notable peaks occurred after the initial emergence of the virus in Denmark during February and March 2020, and again following the spread of the Alpha variant (B.1.1.7) in late 2020. The highest peak occurred after the spread of the Omicron variant in late 2021.

### Coincidental deaths

During most of the pandemic, the 30-day COVID-19 case incidence has been low. However, during winter 2021/22, a combination of widespread transmission and high test activity resulted in the 30-day case incidence rising steeply (Figure 1A).

**Figure 2:**
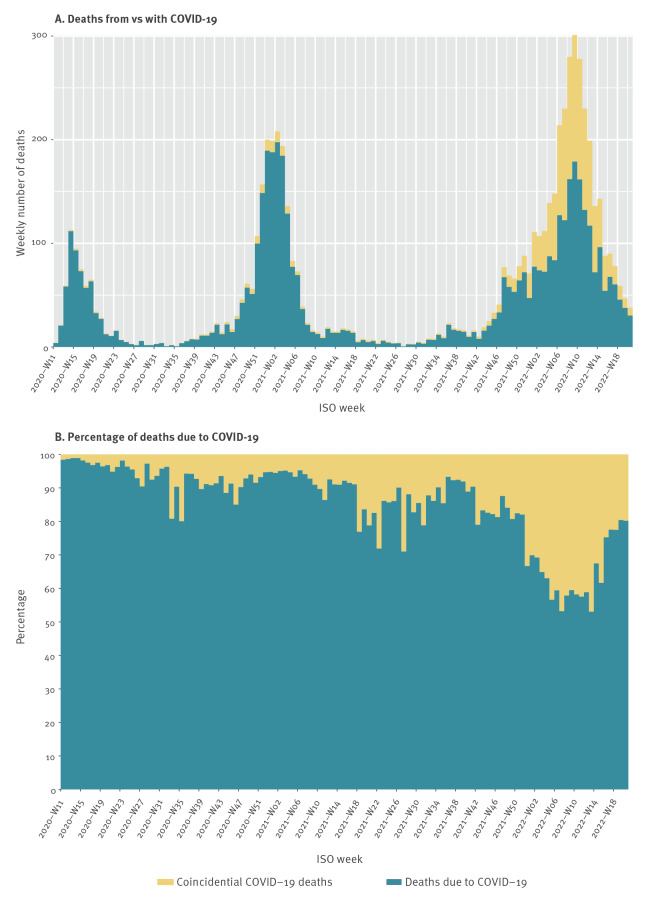
Number of deaths of COVID-19, number of coincidental deaths, and percentage of deaths of COVID-19 among total registered COVID-19 deaths after mathematical correction, Denmark, week 11/2020–week 21/2022

COVID-19: coronavirus disease; ISO: International Organization for Standardization.

The sum of the age-specific estimates is shown in Figure 2A, along with the ratio of COVID-19 unrelated mortality vs all registered COVID-19 mortality (Figure 2B). As depicted in Figure 2B, this method suggests a marked change in the share of coincidental deaths, rising from 10–20% to around 40% of deaths in the period of population-wide spread of the Omicron variant.

### COVID-19 mortality based on death certificates

Among 5,859 30-day COVID-19 case fatalities with a validated cause of death certificate, 3,248 (55%) deaths were classified as a death ‘caused by’ COVID-19, while 2,611 (45%) deaths were classified as a death ‘with’ COVID-19 between the start of the pandemic and 29 May 2022. Pending death certificates made up 504 (8%) of the total 6,363 30-day death count. An additional 475 COVID-19 deaths based on validated death certificates were not reported by the 30-day COVID-19 death count method.

**Figure 3:**
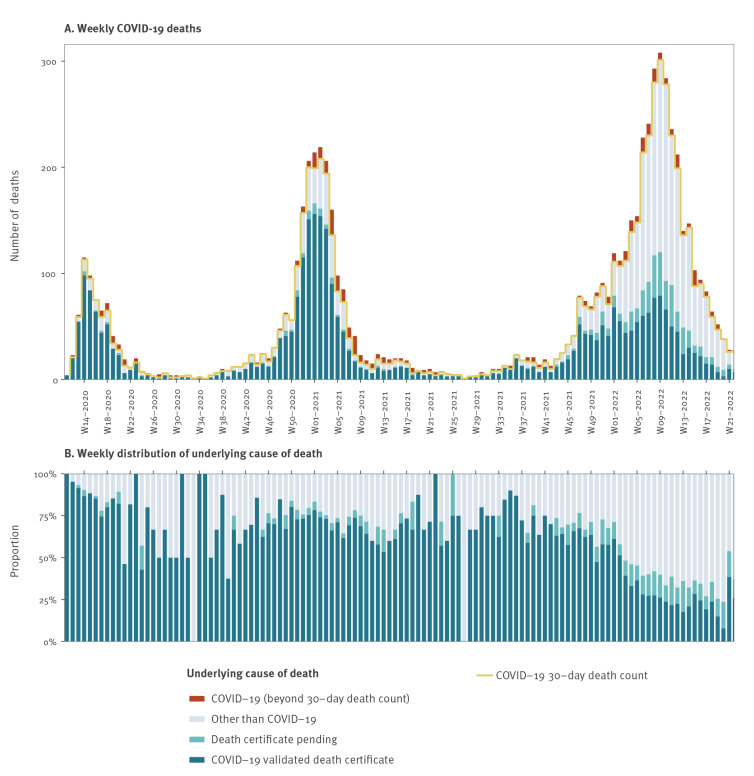
Weekly 30-day deaths from COVID-19 and other causes, by death certificate status, and additional COVID-19 deaths not included in the 30-day COVID-19 death count definition, Denmark, week 11/2020–week 21/2022

Until late 2021, the 30-day COVID-19 case fatalities in general correlated well with the number of COVID-19 deaths reported to the CDR. However, coinciding with the rapid spread of the Omicron variant, the 30-day COVID-19 registered case fatalities increased considerably faster than the number of COVID-19 deaths arising from validated death certificates. This discrepancy rose with rising case numbers, which was also observed during the peak of transmission during winter 2020/21, but back then only to a lesser extent. The share of deaths of COVID-19 (as opposed to with COVID-19) fell from 60–75% before week 1/2022 to 25–35% in the following months (Figure 3). There is still some uncertainty to the last few weeks due to the delay in registration.

COVID-19: coronavirus disease.

### Excess all-cause mortality during COVID-19

**Figure 4:**
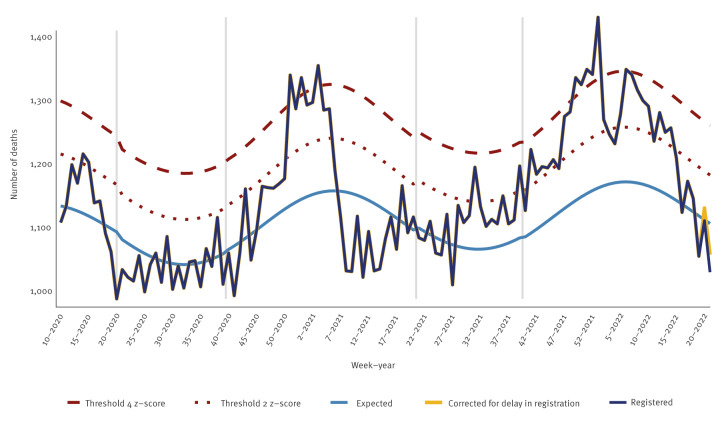
All-cause excess mortality (number of deaths) for all ages, Denmark, week 10/2020–week 21/2022

During the last quarter of 2021, excess all-cause mortality in Denmark was at a stable low-to-moderate level, however with a brief peak of some substantial excess mortality in December 2021, before restrictions against COVID-19 were lifted (Figure 4). In the beginning of 2022, excess mortality fluctuated within the range of moderate excess mortality with an overall downward trend, reaching a normal level from week 13/2022. Notably, the substantial peak in the 30-day death counts observed in January and February of 2022 (Figure 1B) was not reflected in the excess all-cause mortality data.

The grey vertical lines show when data was locked. Data from EUROMOMO [19].

## Discussion

Our retrospective comparative analysis of COVID-19 mortality using various national mortality surveillance sources demonstrates that the mortality burden associated with COVID-19 attenuated after the introduction of the Omicron variant in Denmark, even though mortality numbers reported in international media suggested otherwise. Mortality surveillance is complex in nature and can be vulnerable to bias and other limitations that can be addressed by incorporating alternative methods in the surveillance.

The strong advantage of applying the ‘proxy’ 30-day COVID-19 death count in routine reporting is its timeliness and low resource requirement, given its digitalised and automated generation. It has been disseminated in public reports and dashboards and has also been used by international COVID-19 monitoring sites [6,13]. The 30-day COVID-19 death count served as a valuable tool for public health surveillance and ongoing rapid assessment of COVID-19 severity during the earlier phases of the pandemic but has later proven inadequate and even misleading as the pandemic evolved, causing misunderstandings and confusion in international media. The 30-day COVID-19 death count will not capture deaths that occur later than 30 days after a positive test. Further, COVID-19 deaths in individuals who had not been tested, will not be captured in this surveillance; however, given the high level of testing in Denmark, such ‘additional’ COVID-19 deaths should be rare. These two types of deaths correspond to an increase of merely 7.5% in the number of deaths as identified by the 30-day criterion – and they have occurred throughout the pandemic (see results for weekly COVID-19 deaths by cause of death).

The 30-day criterion also has the important disadvantage that it will incorrectly attribute COVID-19 as the cause of death for every person with a recent positive SARS-CoV-2 test even if the cause of death was in fact unrelated to COVID-19. With the higher incidence of infection and a generally more benign illness caused by the Omicron variant in a national context of high booster vaccination uptake, as is the case in Denmark, a larger fraction of deaths will wrongly be classified as COVID-19 deaths, leading to an overestimation of the COVID-19 mortality burden. Altogether, the 30-day COVID-19 death count therefore represents a trade-off between timeliness and precision when applied for routine national surveillance purposes.

The cause of death registered on the death certificate relies on the physician's individual assessment and may underestimate the impact of COVID-19, in particular when infections with the Omicron variant have a less characteristic clinical presentation and deaths occur outside the hospital setting in people not tested for COVID-19. A small autopsy study from Switzerland with 62 cases found COVID-19 to be the most likely cause of death in five cases where COVID-19 disease was not reported before death [14]. Experiences from other respiratory diseases also suggest that in situations with multiple causes, the primary cause of death tends to be attributed to other causes than the respiratory disease [15]. In contrast, the corrected estimate of COVID-19 deaths includes deaths where COVID-19 was a contributing factor among multiple causes. As such, this method will produce a higher estimated number of COVID-19-related deaths than the number of deaths based on validated death certificates where COVID-19 is exclusively listed as the underlying cause of death.

The excess mortality surveillance is independent of changes in diagnostic testing activity and use of ICD codes and hence provides a more complete and unbiased picture of the total mortality burden of COVID-19. However, it is challenging to infer the cause of any excess mortality in the presence of more than one major public health event, such as in the case of COVID-19 illness and deaths coinciding with seasonal influenza epidemics, which we saw in Denmark in March 2022, with both infections especially affecting the elderly population.

During the steep increase in infections following the introduction of the Omicron variant, which was associated with less severe disease than during earlier phases of the pandemic, the 30-day COVID-19 death count became less accurate, as evidenced by the increased discrepancy with the other methods and data sources used to assess the COVID-19 mortality burden. The COVID-19 deaths based on validated death certificates were less affected during the Omicron-dominated period. Calculations correcting the 30-day COVID-19 death count for coincidental deaths and the low excess mortality seen since late 2021 confirm this change in the COVID-19 mortality pattern. A similar situation has been experienced in other countries, e.g. in the United Kingdom that also lifted restrictions early in 2022 [16] and in South Africa where the Omicron variant was first reported [17] and where reported deaths deviated from the incidence during the Omicron wave [18].

## Conclusion

Our retrospective in-depth analysis of the COVID-19 mortality burden confirms an important shift in COVID-19 mortality following the emergence and spread of the Omicron variant from late 2021 onwards, with a markedly higher proportion of people estimated to have died with, rather than of, COVID-19 as compared with mortality patterns observed earlier in the COVID-19 pandemic. In other words, the mortality related to COVID-19 after the emergence of the Omicron variant has attenuated. We have presented a method for adjusting COVID-19 deaths for coincidental deaths and also showed how different mortality surveillance systems can supplement each other to estimate more correctly the burden of COVID-19 and other diseases. However, it is important to note that even coincidental infections may have contributed to hastening the death in some cases. The evolving COVID-19 pandemic has highlighted the need for an adaptable surveillance system and for ongoing evaluation and comparison of alternative surveillance systems to avoid misunderstandings and ensure correct and unbiased information to the general public and public health authorities.

## Supplementary Data

49000 Supplement
