# Supplementary material for: COVID-19 mortality attenuated during widespread Omicron transmission, Denmark, 2020 to 2022
Source: Euro Surveill. 2023 Jan 19;28(3):2200547. doi: 10.2807/1560-7917.ES.2023.28.3.2200547 (PMC9853946; doi:10.2807/1560-7917.ES.2023.28.3.2200547)
Supplement: Supplement [file 22-00547_FRIIS_Supplement.pdf]

Statistical considerations of the method for assessing the number of deaths due to COVID-19

Disclaimer: This supplementary material is hosted by Eurosurveillance *as supporting information alongside the article COVID-19 mortality attenuated during widespread Omicron transmission in Denmark, on behalf of the authors, who remain responsible for the accuracy and appropriateness of the content. The same standards for ethics, copyright, attributions and permissions as for the article apply. Supplements are not edited by* Eurosurveillance *and the journal is not responsible for the maintenance of any links or email addresses provided therein.*

For population groups with low COVID-19 mortality, our method may give a negative number of deaths due to COVID-19 due to statistical fluctuations. To see how this can occur, we consider the population as subject to two sources of mortality, a general mortality – not related to COVID-19 – and a mortality due to COVID-19. We assume that both types of mortality follow Poisson distributions with intensities λ and η. The population is divided into two groups: those that have tested positive for COVID within the 30-day period and those who have not. If the prevalence of a positive 30-day test is p, the sizes of the two populations are given as pN and $(1-p)N$.

We now consider three random variables:

X_+: the number of non-COVID-19 deaths that have occurred in the test positive population. X_+ will follow a Poisson distribution $P(\lambda pN)$.

X_-: the number on non-COVID-19 deaths that have occurred in the population that have not tested positive. X_- follows a Poisson distribution $P(\lambda(1-p)N)$.

Y: the number of deaths that are attributable to COVID-19. We assume that all deaths will occur among those that have tested positive. Y follows a Poisson distribution $P(\eta pN)$.

Two observations are available: The total number of deaths in the group D irrespective of test-status and the number of death that have occurred in the test-positive group C. We have

$$D = X_+ + X_- + Y$$
$$C = X_+ + Y$$

It is straight forward to see that if the observed values of D and C are denoted d and c then the maximum likelihood estimators for η and λ are

$$\hat{\lambda}pN = \frac{d-c}{1-p}$$
$$\hat{\eta}pN = \frac{c-pd}{1-p}$$

as stated in the main text.

While the positivity of $\hat{\lambda}$ is clearly satisfied, our estimate of $\hat{\eta}$ may become negative as the distribution of

$$\hat{\eta}pN = Y + \left(X_+ - \frac{p}{1-p}X_-\right)$$

has a positive probability of taking negative values. The estimate of the total number of deaths due to COVID-19 ($\hat{\eta}pN$) has two components, Y giving the number of actual COVID-19 deaths (which cannot be observed directly) plus an additional error term

$$Z = X_+ - \frac{p}{1-p} X_-$$

describing the additional statistical variation in the estimate.

We note that the expected value of Z is

$$E(Z) = E(X_+) - \frac{p}{1-p} E(X_-) = \lambda pN - \frac{p}{1-p} \lambda(1-p)N = 0$$

Since X_+ is Poisson distributed $P(\lambda N)$ and X_- follows $P(\lambda(1-p)N)$.

The variance of Z, calculated as

$$Var(Z) = \lambda pN + \left(\frac{p}{1-p}\right)^2 \lambda(1-p)N = \frac{p}{1-p} \lambda N$$

shows the additional uncertainty in the estimate of the number of deaths by COVID-19 that arises due to the use of the 30-day criterion. In particular there is a positive probability of estimating a negative number death due to COVID-19 in the situation where η is small compared to λ (i.e. when COVID-19 death is rare compared to the all-cause mortality) and when p is large.

Negative values of $\hat{\eta}$ and hence negative values of estimated deaths due to COVID-19, does occur in practice for younger age groups.
